# Rosemary supplementation (*Rosmarinus oficinallis* L.) attenuates cardiac remodeling after myocardial infarction in rats

**DOI:** 10.1371/journal.pone.0177521

**Published:** 2017-05-11

**Authors:** Bruna Paola Murino Rafacho, Priscila Portugal dos Santos, Andréa de Freitas Gonçalves, Ana Angélica Henrique Fernandes, Katashi Okoshi, Fernanda Chiuso-Minicucci, Paula S. Azevedo, Leonardo Antonio Mamede Zornoff, Marcos Ferreira Minicucci, Xiang-Dong Wang, Sergio Alberto Rupp de Paiva

**Affiliations:** 1 Internal Medicine Department, Botucatu Medical School–UNESP, Botucatu/SP, Brazil; 2 Department of Biochemistry, Botucatu Biosciences Institute–UNESP, Botucatu/SP, Brazil; 3 Department of Microbiology and Immunology, Botucatu Biosciences Institute–UNESP, Botucatu/SP, Brazil; 4 Nutrition and Cancer Biology Laboratory, Jean Mayer USDA Human Nutrition Research Center on Aging, Tufts University, Boston/MA, United States of America; Scuola Superiore Sant'Anna, ITALY

## Abstract

**Background:**

Myocardial infarction (MI) is one of the leading causes of morbidity and mortality worldwide. Dietary intervention on adverse cardiac remodeling after MI has significant clinical relevance. Rosemary leaves are a natural product with antioxidant/anti-inflammatory properties, but its effect on morphology and ventricular function after MI is unknown.

**Methods and results:**

To determine the effect of the dietary supplementation of rosemary leaves on cardiac remodeling after MI, male Wistar rats were divided into 6 groups after sham procedure or experimental induced MI: 1) Sham group fed standard chow (SR0, n = 23); 2) Sham group fed standard chow supplemented with 0.02% rosemary (R002) (SR002, n = 23); 3) Sham group fed standard chow supplemented with 0.2% rosemary (R02) (SR02, n = 22); 4) group submitted to MI and fed standard chow (IR0, n = 13); 5) group submitted to MI and fed standard chow supplemented with R002 (IR002, n = 8); and 6) group submitted to MI and fed standard chow supplemented with R02 (IR02, n = 9). After 3 months of the treatment, systolic pressure evaluation, echocardiography and euthanasia were performed. Left ventricular samples were evaluated for: fibrosis, cytokine levels, apoptosis, energy metabolism enzymes, and oxidative stress. Rosemary dietary supplementation attenuated cardiac remodeling by improving energy metabolism and decreasing oxidative stress. Rosemary supplementation of 0.02% improved diastolic function and reduced hypertrophy after MI. Regarding rosemary dose, 0.02% and 0.2% for rats are equivalent to 11 mg and 110 mg for humans, respectively.

**Conclusion:**

Our findings support further investigations of the rosemary use as adjuvant therapy in adverse cardiac remodeling.

## Introduction

Myocardial infarction (MI) is one of the leading causes of morbidity and mortality worldwide. According to the 2015 update of A Report From the American Heart Association, approximately 635,000 Americans have a new coronary attack each year[1]. MI can be defined as a focus of necrosis resulting from poor tissue perfusion, with signs and symptoms resulting from cardiac cell death. The death of myocytes initiates a cascade of intracellular signaling, such as inflammation, oxidative stress, reabsorption of necrotic tissue, excessive deposition of collagen, and hypertrophy, that can result in adverse cardiac remodeling. These molecular, cellular and interstitial changes can clinically be manifested as changes in size, mass, geometry and heart function. Cardiac remodeling is an adaptation of the heart to aggression stimuli that may gradually lead to the development of heart failure (HF), responsible for the increased mortality after MI[2, 3].

Many factors can participate on MI pathophysiology. MI is started by myocardial ischemia and it is associated with increased generation of reactive oxygen species (ROS)[4]. In experimental studies, oxidative stress is identified as a major factor for the development of cardiac hypertrophy[5]. Oxidative stress can also activate the production of inflammatory cytokines such as tumor necrosis factor-α (TNF-α), IL-1β and IL-6[6], triggering inflammatory pathways, fibrosis and cell death[5]. ROS and cytokines also contribute to the activation of matrix metalloproteinases (MMPs) and collagen deposition that might lead to structural changes in the heart [6, 7].

Because oxidative stress can play a central pathophysiological role in cardiac remodeling after MI[4, 7], antioxidant supplements are beneficial after injury to the myocardium[8, 9]. In this context, the antioxidant properties of natural products have been examined[10]. Rosemary (*Rosmarinus oficinallis* Linn) is a popular culinary spice, but it is also known as a medicinal herb and a natural conservative in the food industry, with one of the highest levels of antioxidant compounds[11, 12]. Many compounds have been isolated from rosemary, including flavones and diterpenes. The phenolic diterpenes carnosic acid and carnosol are the major bioactive compounds in rosemary leaves related to the antioxidant activity [12, 13]. Rosmarinic acid is a caffeic acid ester with antioxidant and anti-inflammatory activity[14, 15]. *In vitro* studies described that rosemary compounds suppressed IL-β and TNF-α, and increased glutathione peroxidase (GSH-Px) and superoxide dismutase (SOD) activity in different models[16–18]. *In vivo*, rosemary extract supplementation improved the oxidative stress status in the heart of aged rats[19, 20]. Furthermore, rosmarinic acid reduced myocardial damage blood pressure in hypertensive rats fed a high fructose diet[15] and protected the heart against cardiac dysfunction and fibrosis after MI in rats[21]. However, little is known about the effect of rosemary leaves intake in morphology and ventricular function after myocardial injury. To our knowledge, the protective potential of rosemary has predominantly been studied in rosemary extract and/or its constituents, and information about rosemary intake as a whole food is limited. Evidence shows that focusing on an approach based on foods and dietary patterns instead of individual nutrients improves cardiometabolic health[22, 23]. Thus, the aim of the present study was to evaluate the effect of rosemary leaves dietary supplementation on cardiac remodeling after myocardial infarction.

## Methods

### Study design

All experiments and procedures were performed in accordance with the National Institute of Health’s Guide for the Care and Use of Laboratory Animals and with the Ethical Principles in Animal Experimentation adopted by the Brazilian College of Animal Experimentation[24]. The study protocol (838/10) was submitted and approved by the Botucatu Medical School Animal Research Ethics Committee.

Male Wistar rats weighing 200 to 250 g were used in this study. MI was conducted by coronary artery ligation, as previously described[2, 25]. In brief, rats were anesthetized with ketamine (70 mg/kg) and xylazine (1 mg/kg), and after left thoracotomy, the heart was exteriorized by lateral compression of the thorax. The left atrium was retracted to facilitate ligation of the left coronary artery with wired polyvinyl (5–0 Ethicon). The left coronary artery was ligated approximately 2 mm between the border of the left atrium and the pulmonary outflow tract. The heart was then replaced in the thorax, the lungs were inflated by positive pressure, and thoracotomy closed. A sham group, in which animals were submitted to surgery but without coronary occlusion, was also created. After surgery rats were housed in a temperature-controlled room (24°C) with a 12-h light/12-h dark cycle. Water and food was supplied *ad libitum* after the procedure.

**Fig 1:**
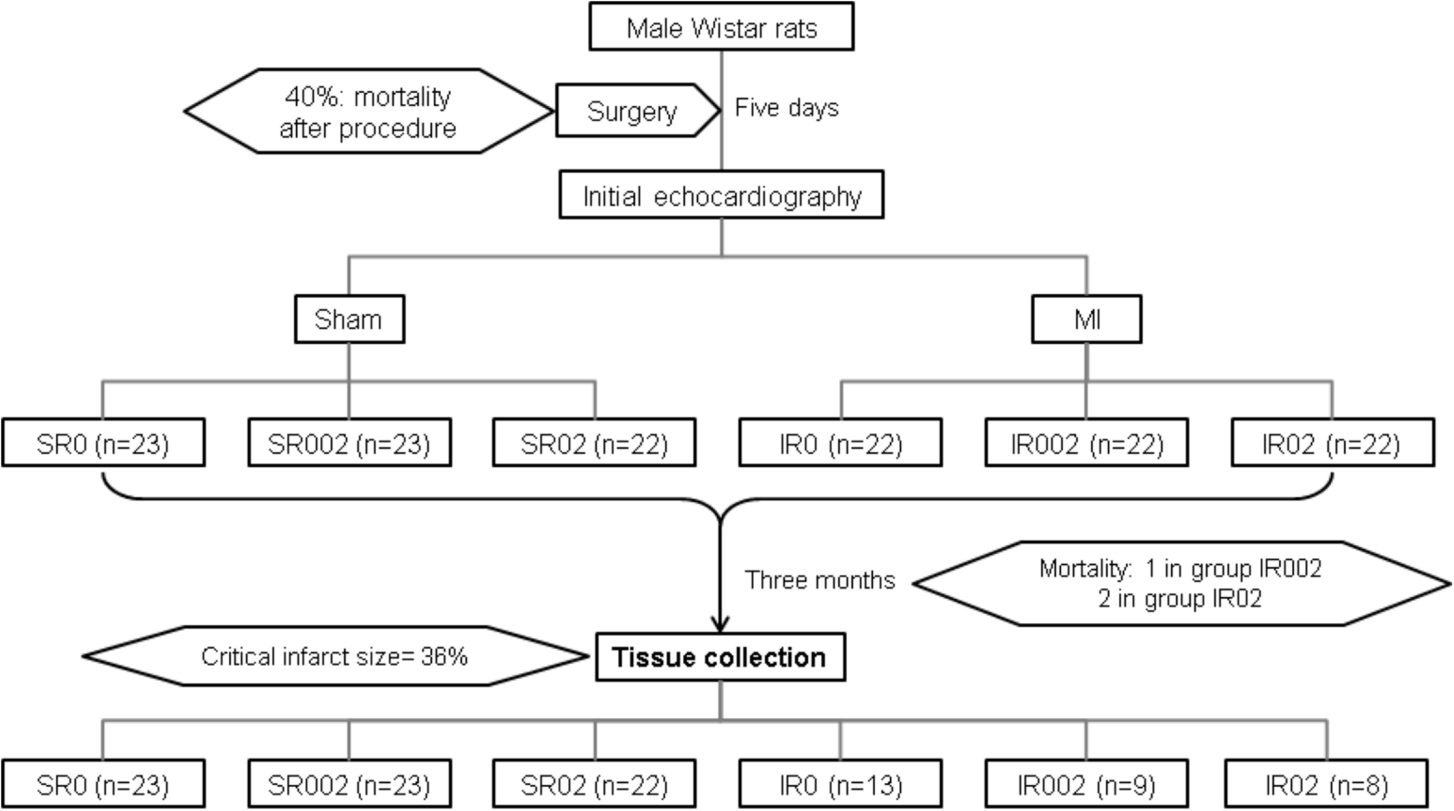
Study design.

Two days after surgery, survivors were assigned to one of the six groups: 1) group SR0, sham animals fed standard chow only (n = 23); 2) group SR002, sham animals fed standard chow with 0.02% of rosemary leaves (n = 23); 3) group SR02, sham animals fed standard chow with 0.2% of rosemary leaves (n = 22); 4) IR0, infarcted animals fed standard chow only (n = 13); 5) IR002, infarcted animals fed standard chow with 0.02% of rosemary leaves (n = 8); and 6) IR02, infarcted animals fed standard chow with 0.2% of rosemary leaves (n = 9) (Fig 1). Treatment began 48 h after surgery because during this period mortality may be related to bleeding, pneumothorax, and anesthesia rather than to the infarction or treatment. Rosemary supplementation was provided for 90 days.

### Rosemary supplementation

Nuvilab chow (Nuvital®) was used for all experiments. Chow was initially chopped for the later addition of rosemary leaves. Fresh rosemary leaves were purchased in 2011 from Companhia de Entrepostos e Armazéns Gerais de São Paulo (CEAGESP), state of São Paulo, Brazil and were identified by a trained dietitian. The leaves were oven dried for 48 hours at a temperature of 50°C, ground in a domestic mixer (Walita, São Paulo, Brazil) for 30 seconds, and sieved using sieves of the Standard Tyler 32 (Bertel Industries, São Paulo, Brazil). Ground particles were stored under vacuum and maintained in a domestic freezer (Brastemp, São Paulo, Brazil) below –10°C. Rosemary powder was added to the crushed chow, and the mixture was pelletized. Food intake of all animals was measured every 24 hours. The mean daily intake for each rat was calculated. Rosemary supplementation doses of 0.02% and 0.2% were chosen based on the study of Posadas et al. (2009)[19].

### Measurement of systolic arterial pressure

Systolic arterial pressure measurement was performed 2 weeks (corresponding to 2.5 months of rosemary supplementation) before euthanasia by tail plethysmograph, as described previously[26].

### Echocardiographic study

After three months of supplementation, rats were weighed and evaluated by a transthoracic echocardiographic exam, as previously described[27, 28]. All measurements were made by the same observer blinded to individual animal treatments and according to the American Society of Echocardiography/European Association of Echocardiography[29].

After the echocardiographic study, the animals were euthanized with large dose of pentobarbital, and their hearts were removed. Left ventricle (LV) was isolated and LV samples were immediately frozen and stored at -80°C. One transverse section of the LV was separated and fixed in 10% buffered formalin and then was embedded in paraffin for histological study.

### Morphometric analysis

Five-micrometer-thick sections were stained with hematoxylin and eosin (HE) for cardiomyocyte cross-sectional area (CSA) determination and with Pircrosirius red for Interstitial collagen fraction (ICF) and infarction size calculations. All animals were included in the morphometric analysis. First infarction size was calculated. To calculate infarction size, lengths of the infarcted and the viable muscle for both endocardial and epicardial circumferences were determined by planimetry, and then calculated by dividing endocardial and epicardial circumferences of the infarcted area by total epicardial and endocardial ventricular circumferences[28]. Measurements were performed on midventricular slices (5–6 mm from the apex), under the assumption that the left midventricular slice showed a close linear relation with the sum of the area measurements from all heart slices[30]. After infarction size calculation, infarcted animals with less than 36% of LV infarcted area were excluded of further analysis[31, 32]. Minicucci et al. (2011) showed that the infarct size cut-off value to induce cardiac remodeling in rats should be 36% of LV area[31].

The CSA measurements were obtained from at least 40 digital images(400 × magnification) with a digital pad, and the selected cells were transversely cut so that the nucleus was in the center of the myocyte[33, 34]. The CSA was considered to evaluate heart hypertrophy. ICF was determined in remote cardiac areas free from MI from at least 20 digital images (400 × magnification).

All images were collected with a video camera attached to a Leica microscope; the images were analyzed with the Image-Pro Plus 3.0 software program (Media Cybernetics; Silver Spring, MD).

### Cytokine production

Tumor necrosis factor-α (TNF-α), IFN-γ and IL-10 concentrations in LV samples were determined by ELISA according to the manufacturer’s instructions (R&D Systems, Minneapolis, MN).

### MMP-2 and TIMP-1 evaluation

Matrix metalloproteinase (MMP)-2 activity was determined in LV samples by zimography, as previously reported[34, 35]. TIMP-1 levels were evaluated by ELISA according to the manufacturer’s instructions (R&D Systems, Minneapolis, MN

### Lipid hydroperoxide, antioxidant and energy metabolism enzymes

Eight LV samples of each experimental group were used for measurements of total protein and lipid hydroperoxide (LH) concentration and for enzyme activity determinations. Glutathione peroxidase (GSH-Px, E.C.1.11.1.9), superoxide dismutase (SOD, E.C.1.15.1.1) and catalase (CAT, E.C.1.11.1.6) activity was assessed as previously specified[36, 37]. Cardiac energy metabolism was assessed by β-hydroxyacyl coenzyme-A dehydrogenase (OHADH, E.C.1.1.1.35.), lactate dehydrogenase (LDH, E.C.1.1.1.27), citrate synthase (CS; E.C.4.1.3.7.), Complex I (NADH:ubiquinone oxidoreductase), Complex II (succinate dehydrogenase), and ATP synthase (EC 3.6.3.14) activities, as previously described[37, 38]. Spectrophotometric determinations were performed with a Pharmacia Biotech spectrophotometer UV/visible Ultrospec 5000 with Swift II Application software (Cambridge, England, UK) at 560 nm. All reagents were purchased from Sigma (St. Louis, Missouri, USA).

### Western blot analysis

Briefly, left ventricular samples were extracted using RIPA Buffer to detect heme-oxygenase-1 (HO-1), caspase-3, Bcl2 and peroxisome proliferator-activated receptor-α coactivator (PGC)-1α expression. To determine nuclear erithroid factor 2 (Nrf-2), LV samples were extracted with Nuclear Extraction Buffer[39]. The following primary antibodies were used: HO-1-1 to heme oxygenase-1; ab13248 (Abcam Inc, Cambridge); Nrf2: C-20, rabbit Immunoglobulin G (Santa Cruz Biotechnology Inc, Europe); Cleaved Caspase-3 (Asp175) (5A002E) Rabbit mAb (Cell Signalling Technology Inc., USA); Bcl2 sc 492 IgG rabbit monoclonal (Santa Cruz Biotechnology, Inc, Europe); and PGC-1α Antibody H-300: sc-13067 (Santa Cruz Biotechnology, Inc, Europe). GAPDH (GAPDH (6C5), mouse monoclonal IgG1, (Santa Cruz Biotechnology, Inc., Europe, sc 32233) was used for normalization.

### Statistical analysis

Data are presented as the mean±SEM. The results were tested for both normality (Kolmogorov–Smirnov test) and equal variance before statistical analyses, and all data passed these tests. Data were analyzed by 2-factor ANOVA, therefore this analysis gives three p values: 1) factor one: presence of myocardial infarction (I); 2) factor two: rosemary content (R); and 3) interaction between factors I and R. When an interaction was found to be significant, the mean values were compared using Holm-Sidak post hoc analysis. If an interaction was not found, the separated factors were analysed (marginal data). A χ^2^ test was used to evaluate mortality between infarcted animals. One-factor ANOVA was used to analyze infarction size in infarcted groups. Differences were to be considered statistically significant if *P*<0.05. Graphs and statistical analyses were performed using SigmaPlot for Windows version 12.0 (Systat Software Inc. San Jose, CA).

## Results

### Survival, food intake, body weight and systolic arterial pressure

The mortality rate within 48 hours after infarction was 43%. No death in the Sham animals was observed. In the infarcted groups, two animals in both the IR0 and IR02 groups died, and one animal in the IR002 group died (p = 0.765). No difference was observed in infarction size between infarcted groups (Table 1).

**Table 1:** Food intake, body weight, infarction size, echocardiographic and morphometric studies in Sham and infarcted rats with and without rosemary supplementation.

	SHAM groups			Myocardial infarction groups			p values		
	SR0	SR002	SR02	IR0	IR002	IR02	p (I)	p (R)	p (IxR)
**Food intake (g)**	25.8±0.2	24.6±0.2	25.7±0.2	25.4±0.2	25.0±0.2	25.5±0.2	0.187	0.101	0.143
**Weight gain (g)**	143±8.2	157±8.0	148±8.6	152±8.8	156±8.8	159±10	0.385	0.552	0.773
**Infarction size (%)**	-	-	-	41.9±4.5	40.1±4.2	43.6±4,7	-	0.244	-
**LVDD/BW (mm/kg)**	17.9±0.41	17.1±0.39	17.9±0.29	24.6±0.74	24.2±0.68	24.1±0.93	**<0.001**	0.504	0.582
**E wave (cm/s)**	75.7±1.5	77.2±2.1	78.9±1.8	98.4±5.5	87.5±8.5	92.7±9.4	**0.014**	0.482	0.246
**Diastolic area (mm**^**2**^**)**	44.4±1.6	44.5±1.8	43.3±1.6	92.2±3.9	91.7±6.9	85.2±4.5	**<0.001**	0.359	0.557
**FAC (%)**	73.3±0.9	74.3±1.2	75.6±1.0	27.0±2.3	28.2±1.5	27.2±3.5	**<0.001**	0.697	0.757
**Ejection fraction**	0.91±0.01	0.92±0.01	0.93±0.01	0.47±0.02	0.49±0.02	0.46±0.02	**<0.001**	0.571	0.612
**E/E’ ratio**	19.1±0.6	19.6±0.9	18.8±0.6	24.4±1.7	25.6±2.8	23.6±2.6	**<0.001**	0.898	0.523

I: infarction; S: Sham; R: Rosemary; R0: no supplementation; R002: 0.02% of rosemary supplementation; R02: 0.2% of rosemary supplementation; Weight gain: Final body weight–initial body weight (g); BW: body weight; LVDD/BW: left ventricular diastolic diameter indexed for body weight; E wave: peak velocity of early ventricular filling; FAC: fractional area change; E/E’ ratio: early diastolic mitral inflow velocity to early mitral annular velocity ratio. Data are expressed as the mean ± SEM. Bold numbers represent the significant effects that were considered.

**Fig 2:**
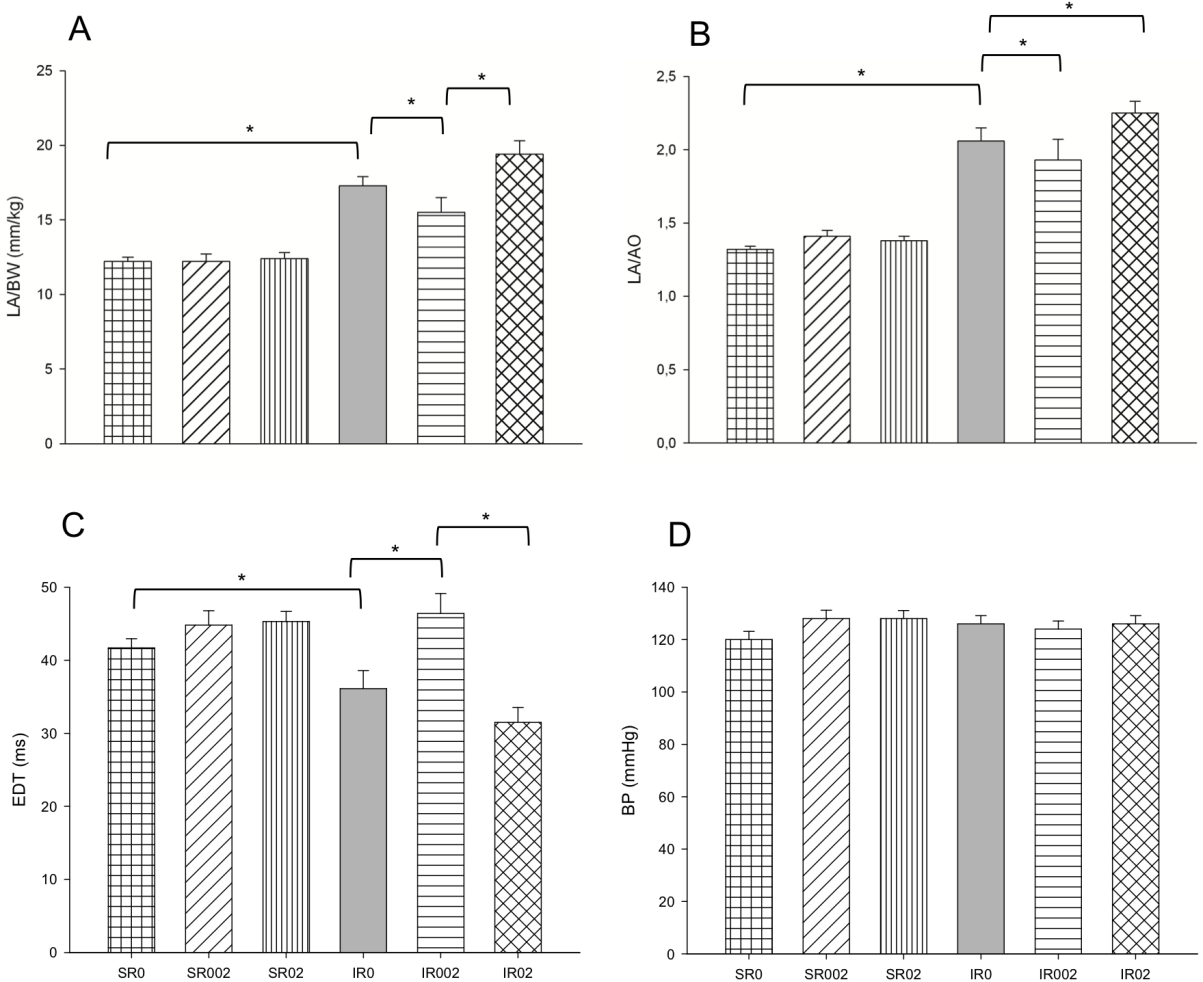
Echocardiographic study and blood systolic pressure in Sham and infarcted rats with and without rosemary supplementation. A. LA/BW: left atrial diameter indexed for body weight (p = 0.001); B. LA/AO: left atrial diameter indexed for aortic diameter (p = 0.024); C. EDT: E wave deceleration time (p = 0.004); D. BP: blood systolic pressure (p = 0.343). Data are expressed as the mean ± SEM. Asterisks (*) represent significant difference between groups (p<0.05). Sample size: SR0 = 23; SR002 = 23; SR02 = 22; IR0 = 13; IR002 = 8; and IR02 = 9.

No difference was observed for food intake, weight gain (Table 1) and systolic arterial pressure (Fig 2D) among all groups.

### Effect of MI in rat hearts

**Fig 3:**
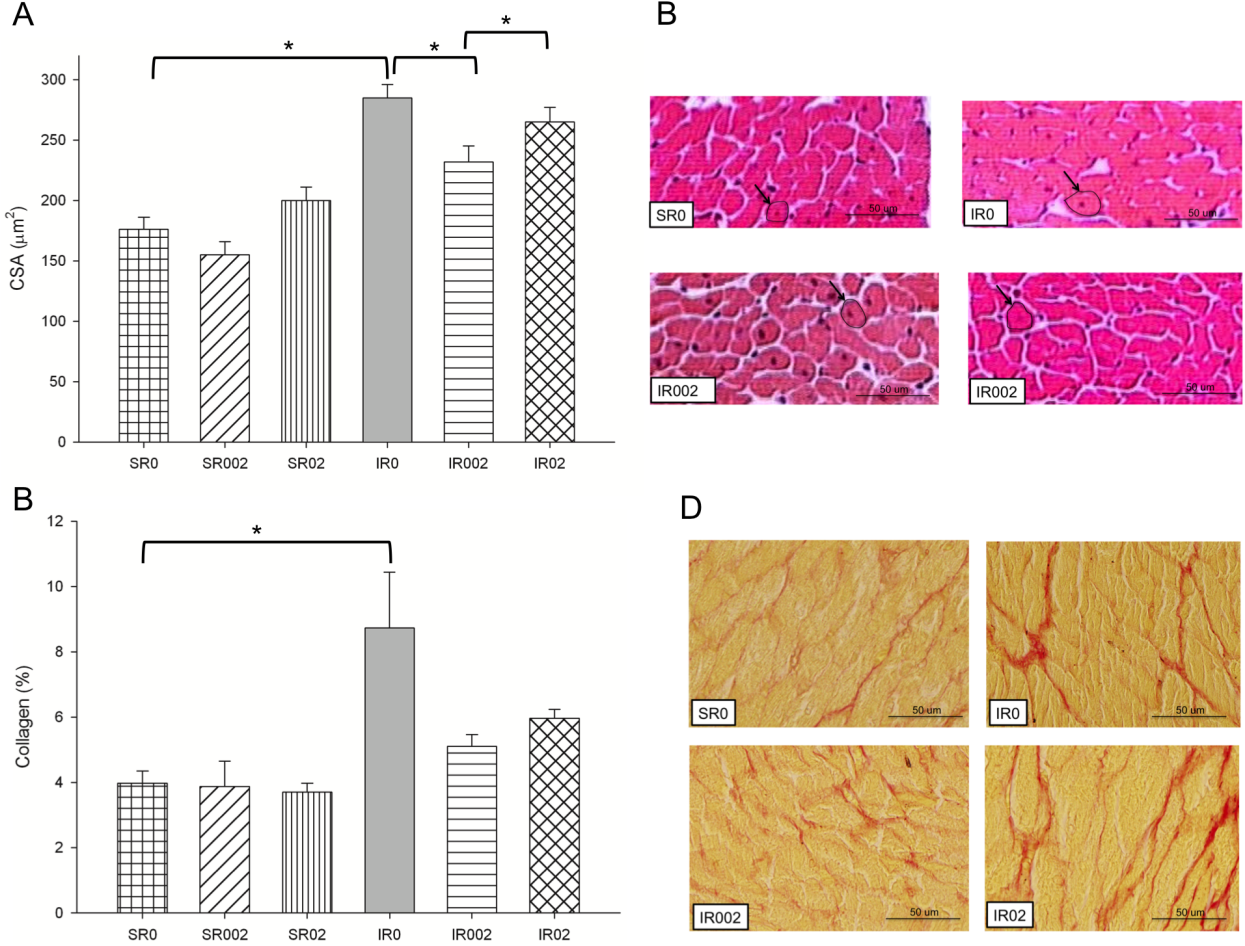
Morphometric study in Sham and infarcted rats with and without rosemary supplementation. A. CSA: cardiomyocyte cross-sectional area (p<0.001); B. Microscopic images of CSA H&E stained (evidenced with arrows) of groups SR0, IR0, IR002 and IR02; C. % collagen: p = 0.035; D. Microscopic images of myocytes Picrosirius red stained for collagen (red marks in images) of SR0, IR0, IR002 and IR02. Data are expressed as the mean ± SEM. Asterisks (*) represent significant difference between groups (p<0.05). Sample size: SR0 = 23; SR002 = 23; SR02 = 22; IR0 = 13; IR002 = 8; and IR02 = 9.

MI led to adverse cardiac remodeling. Regarding morphological data, MI led to higher left ventricular end-diastolic diameter adjusted for body weight, higher diastolic area (Table 1) and higher left atrium (Fig 2A and 2B), CSA (Fig 3A and 3B) and percentage of collagen (Fig 3C and 3D). MI impaired diastolic heart function, as shown by increased shorter E wave deceleration time (EDT) (Fig 2C) and increased E/E' ratio (Table 1), and systolic function showed by lower fractional area change (Table 1).

**Fig 4:**
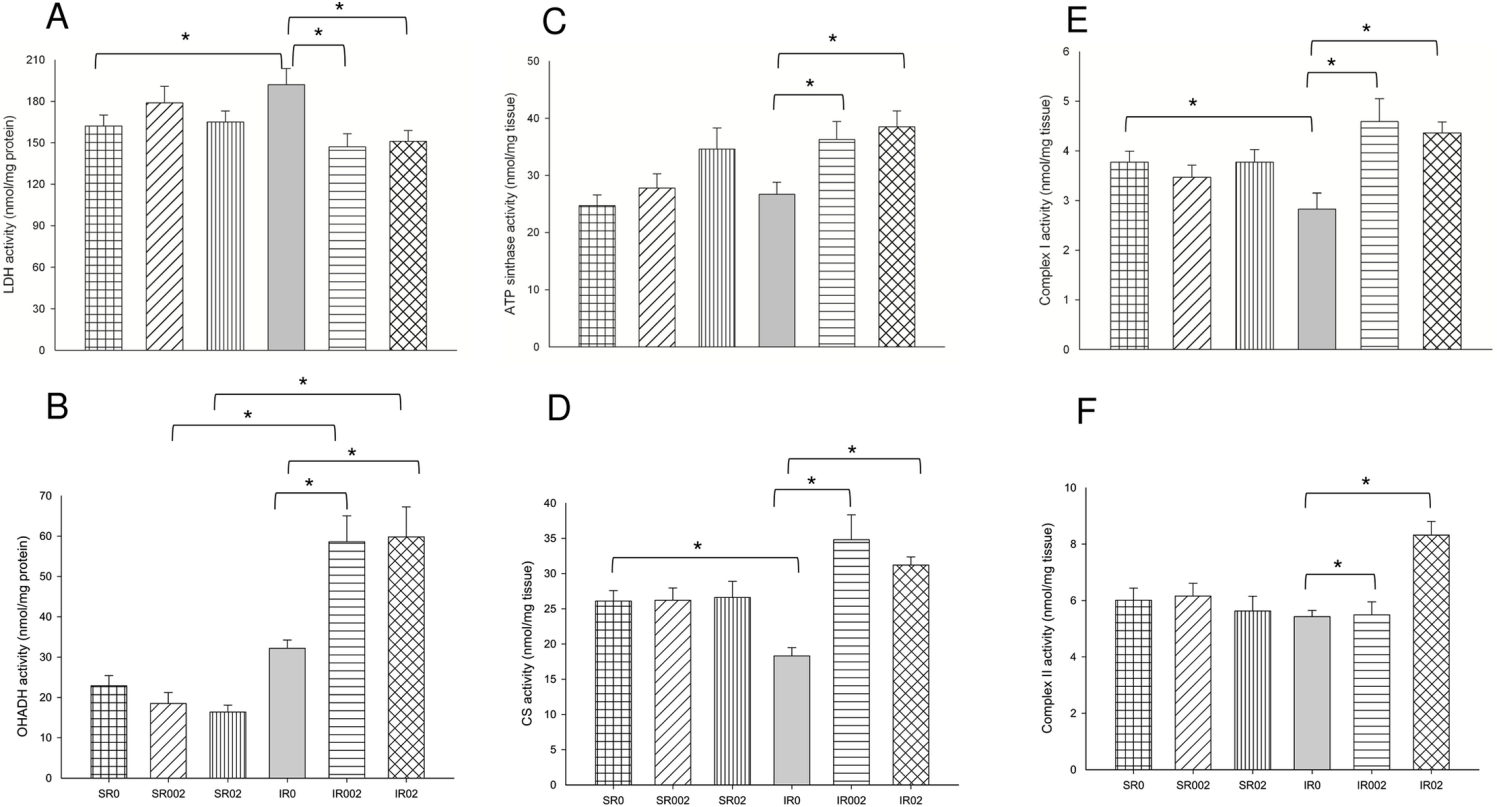
Energy metabolism enzymes in Sham and infarcted rats with and without rosemary supplementation. LDH activity: lactate dehydrogenase activity (p = 0.014); CS activity: citrate synthase activity (p<0.001); OHADH activity: 3-hydroxyacyl coenzyme-A dehydrogenase activity (p<0.001); ATP sinthase activity (p = 0.039); Complex I activity: p = 0.004; Complex II activity: p<0.001. Data are expressed as the mean ± SEM. Asterisks (*) represent significant difference between groups (P<0.05). Sample size: SR0 = 8; SR002 = 8; SR02 = 8; IR0 = 10; IR002 = 7; and IR02 = 8.

**Fig 5:**
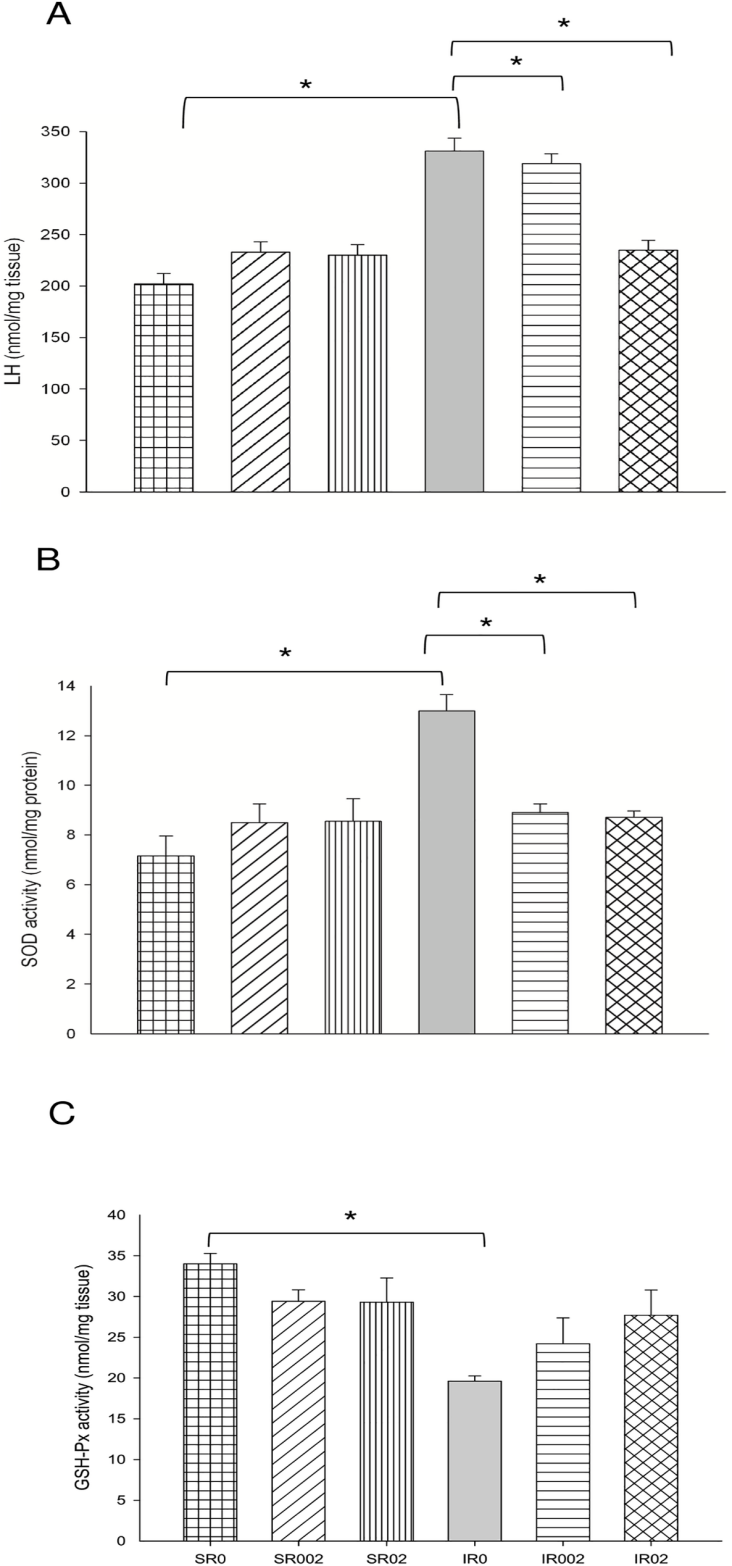
Oxidative stress enzymes in Sham and infarcted rats with and without rosemary supplementation. LH: lipid hydroperoxide concentration (p<0.001); SOD activity: superoxide dismutase activity (p<0.001); GSH-Px activity: glutathione peroxidase activity (p = 0.031). Data are expressed as the mean ± SEM. Asterisks (*) represent significant difference between groups (P<0.05). Sample size: SR0 = 8; SR002 = 8; SR02 = 8; IR0 = 10; IR002 = 7; and IR02 = 8.

**Fig 6:**
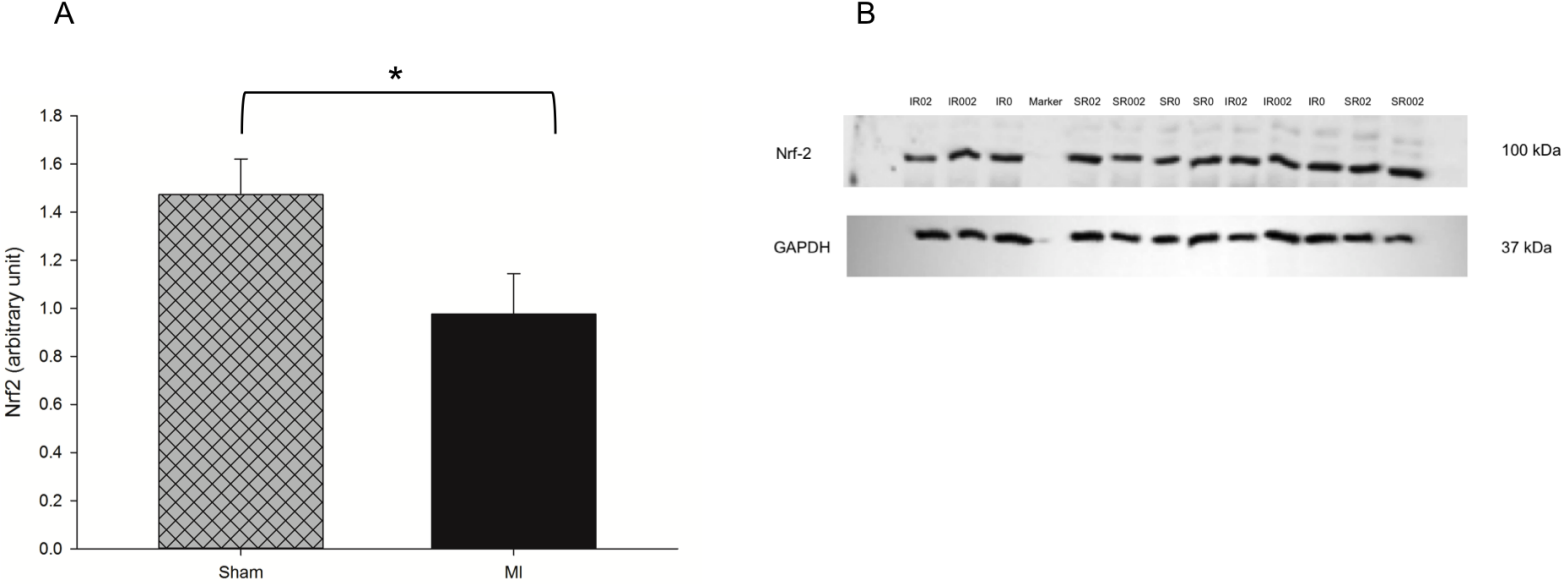
Nrf2 expression in Sham and infarcted rats by Western blot. A. Nrf2 expression; B. representative western blot of Nrf2 expression. Nrf2: nuclear erithroid factor 2; GAPDH glyceraldehyde-3-phosphate dehydrogenase. Since no interaction between factors was observed, the effect of myocardial infarction is represented. Data are expressed as the mean ± SEM. Asterisks (*) represent significant difference between groups. Sample size: SR0 = 10; SR002 = 10; SR02 = 10; IR0 = 10; IR002 = 8; and IR02 = 9.

A greater oxidation of carbohydrates and impaired energy metabolism was observed as shown by higher activity of LDH (Fig 4A and S2 Table) and ATP synthase (Fig 4C and S2 Table), and lower activity of CS (Fig 4D and S2 Table) and Complex I (Fig 4E and S2 Table). MI also increased oxidative stress, as presented with higher LH concentration (Fig 5A and S2 Table) and SOD activity (Fig 5B and S2 Table), lower GSH-Px activity (Fig 5C and S3 Table), and lower expression of Nrf-2 (Fig 6A and 6B). No difference was observed for HO-1 (S3 Table and S1 Fig) and PGC-1α expression (S3 Table and S2 Fig).

Higher intermediate and total MMP-2 activity (S4 Table and S3 Fig), lower IL-10, TNF-α and INF-γ concentration (S4 Table), and lower Bcl2 expression (S4 Table and S4 Fig) was observed after MI. No difference was observed for caspase-3 expression (S4 Table).

### Effect of rosemary supplementation after MI

Rosemary supplementation improved diastolic function (lower left atrial diameter [Fig 2A and 2B and S1 Table] and higher EDT [Fig 2C and S1 Table]) and reduced hypertrophy (Fig 3A and S1 Table) after MI.

Morphologic and functional changes were accompanied by increased β-oxidation of fatty acids, reduced lactate oxidation and improved respiratory chain activity, as shown by lower LDH activity (Fig 4A and S2 Table), and higher CS (Fig 4C and S2 Table), OHADH (Fig 4E and S2 Table), ATP synthase (Fig 4B and S2 Table) and Complex I (Fig 4D and S2 Table) activities. Rosemary supplementation also decreased oxidative stress, with lower concentration of LH (Fig 5A and S2 Table) and SOD activity (Fig 5B and S2 Table). No differences were observed for GSH-Px (Fig 5C and S2 Table) and catalase (S1 Table) activities, and Nrf-2, HO-1 and PGC-1α expression (S3 Table and S3 Fig).

Differences among the doses were also observed in diastolic function and hypertrophy. Group IR002 presented lower left atrium (Fig 2A and 2B and S1 Table) and CSA (Fig 3A and S1 Table). Group IR02 presented higher Complex II activity (Fig 4F and S2 Table).

No difference was observed for percentage of collagen with rosemary supplementation (Fig 3B and S1 Table), cytokines concentration, MMP-2 activity (S4 Table and S3 Fig), caspase-3 and Bcl2 expression (S4 Table and S4 Fig).

## Discussion

The aim of the present study was to analyze the influence of rosemary supplementation of rat chow on adverse cardiac remodeling after myocardial infarction. Cardiac remodeling has significant clinical relevance as it can lead to complex changes in ventricular architecture, potentially evolving into chronic heart failure [7, 40]. For this reason, several strategies have been used to mitigate this process, including compounds found in rosemary leaves[17, 41]. It is well known that lifestyle factors, including nutrition, play a key role in the etiology of Cardiovascular Diseases (CVD), evidencing the importance to promote health diet including selected foods rather than individual nutrients[23, 42]. In this context, the use of rosemary leaves instead of one isolated compound might lead to a synergistic effect, since adjuvant substances in the plant might enhance the activity of benefic components[43]. To our knowledge, this is the first study that assessed the supplementation with whole rosemary leaves on the morphology and function of the infarcted heart.

Myocardial infarction in the rat is an ideal model to study adverse cardiac remodeling post-infarction[44]. Cardiac remodeling can be defined as molecular, cellular, and interstitial modifications that are clinically manifested as changes in size, mass, geometry and heart function after cardiac injury. Heart dysfunction is the final sign of cardiac remodeling and is an important prognostic factor after MI, increasing the risk of death[45, 46]. Diastolic dysfunction after MI refers to mechanical and functional abnormalities during relaxation and filling of the LV and is associated with hypertrophy and increased LA[47]. In the present study, rosemary supplementation after MI improved diastolic function, LV hypertrophy and LA diameters, evidencing the protective effect of rosemary leaves in the heart. Our results are in accordance with previous studies describing improved cardiac function in cardiac injury models by doxorubicin and ischemia/reperfusion after treatment with compounds found in rosemary[48, 49].

The improvement of hypertrophy and diastolic function observed after rosemary intake was associated with changes in energy metabolism and decreased oxidative stress after MI in the present study. As clinical manifestations can be the result of changes to the heart’s cellular and molecular components[3], energy metabolism and antioxidant pathways could represent the mechanisms of action of rosemary leaves supplementation. In injury situations such as MI, the preferential use of fatty acids that are observed in the normal hearts may be shifted for glucose use[50, 51]. In this case, the heart starts to form large amounts of lactate, increasing anaerobic metabolism of carbohydrates and LDH activity[52, 53]. Rosemary supplementation after MI led to higher fatty acid oxidation and respiratory chain improvement, similar to the energy metabolism of normal hearts.

In addition to metabolic changes, oxidative stress and redox signaling are important contributors to cardiac remodeling[54]. Increased oxidative stress and cardiac oxidation has also been associated with diastolic dysfunction[47]. The toxic effects of ROS can be prevented in part by the antioxidant enzyme system including GSH-Px, SOD and catalase[55]. SOD is considered the first line of defense in protecting the mitochondria against deleterious effects of increased superoxide production, as described in cardiac remodeling and HF[56] and observed in the present study. Our results showed that rosemary supplementation decreased oxidative stress, which is in agreement with previous studies reporting antioxidant effect of rosemary and its compounds[11, 19, 57]. Rosemary can mimic SOD by removing superoxide radicals[58], which could also explain the lower enzyme activity in cardiac tissue of the supplemented groups. No effect of rosemary was observed on Nrf-2 and HO-1 expression, different from what we expected and from previous reports showing Nrf2 activation after treatment with rosemary compounds[59, 60]. One possible explanation is that in some pathological situations, as in hypoxia, Nrf-2 does not increase. Regulatory mechanisms such as the E3 ubiquitin (Siah2) protein ligase 2, which is activated in hypoxia, binds to Nrf-2 and increases its degradation, preventing it from acting on AREs and increasing the expression of antioxidant proteins[61].

In the present study, rosemary doses led to different effect in heart diastolic function and hypertrophy, similar to a J-shape response. The smallest dose of rosemary supplementation caused greater improvement in post-MI heart function. [62]. Forman and collaborators (2014) describes that antioxidants in fruits and vegetables maintain a cellular defense and adaptation response. The authors exemplifies that supplementation of phytochemicals to levels that exceed saturation of the antioxidant system will hardly exert any beneficial effect, by a mechanism that can be called “para-hormesis”[62]. In the present study, 0.2% of rosemary supplementation led to a greater antioxidant response (lower LH and higher antioxidant enzymes activities) which could impair the cellular healthy response signaling by ROS[63]. Also our finding of higher Complex II after 0.2% rosemary supplementation could indicate the para-hormesis mechanism. In the mithocondria, Complex II is part of the antioxidant system by controlling the ubiquinone pool and superoxide scavenging activity of the respiratory chain (RC). Complex II might also be activated upon reduction of the RC[64]. So in the present study, the supplementation with the highest dose of rosemary might have lost the protective effect exhibited with 0.02% of rosemary.

The limitation of the present study lies in its experimental type, not allowing us to extrapolate the findings to humans. However, it is important to highlight the clinical relevance of the results described. Our findings support further investigations of rosemary use as adjuvant therapy in adverse cardiac remodeling. Regarding rosemary dose in the present study, 0.02% and 0.2% in the rat chow is equivalent to 11 mg/day and 110 mg/day in humans, respectively[65].

In conclusion, dietary rosemary supplementation attenuated adverse cardiac remodeling caused by myocardial infarction in rats. The mechanism could involve improved energy metabolism and reduced oxidative stress. Rosemary supplementation may serve as a promising approach to attenuate adverse cardiac remodeling after MI.

## Supporting information

S1 Fig Western blot for HO-1 adjusted by glyceraldehyde-3-phosphate dehydrogenase (GAPDH). I: infarction; S: Sham; R: Rosemary; R0: no supplementation; R002: 0.02% of rosemary supplementation; R02: 0.2% of rosemary supplementation. Sample size: SR0 = 10; SR002 = 10; SR02 = 10; IR0 = 10; IR002 = 8; and IR02 = 9.
(TIF)

S2 Fig Western blot for PGC1-α adjusted by glyceraldehyde-3-phosphate dehydrogenase (GAPDH). I: infarction; S: Sham; R: Rosemary; R0: no supplementation; R002: 0.02% of rosemary supplementation; R02: 0.2% of rosemary supplementation. Sample size: SR0 = 10; SR002 = 10; SR02 = 10; IR0 = 10; IR002 = 8; and IR02 = 9.
(TIF)

S3 Fig Zimography picture of metalloproteinase-2 (72 kDa). I: infarction; S: Sham; R: Rosemary; R0: no supplementation; R002: 0.02% of rosemary supplementation; R02: 0.2% of rosemary supplementation. Sample size: SR0 = 10; SR002 = 10; SR02 = 10; IR0 = 5; IR002 = 5; and IR02 = 4.
(TIF)

S4 Fig Western blot for Bcl-2 and Caspase-3 adjusted by glyceraldehyde-3-phosphate dehydrogenase (GAPDH). I: infarction; S: Sham; R: Rosemary; R0: no supplementation; R002: 0.02% of rosemary supplementation; R02: 0.2% of rosemary supplementation. Sample size: SR0 = 10; SR002 = 10; SR02 = 10; IR0 = 10; IR002 = 8; and IR02 = 9.
(TIF)

S1 Table Food intake, body weight, infarction size, echocardiographic and morphometric studies in Sham and infarcted rats with and without rosemary supplementation. I: infarction; S: Sham; R: Rosemary; R0: no supplementation; R002: 0.02% of rosemary supplementation; R02: 0.2% of rosemary supplementation; BW: body weight; LVDD/BW: left ventricular diastolic diameter indexed for body weight; AO: aortic diameter; LA: left atrial diameter; LA/BW: left atrial diameter indexed for body weight; LA/AO: left atrial diameter indexed for aortic diameter; E wave: peak velocity of early ventricular filling; FAC: fractional area change; EDT: E wave deceleration time; E/E’ ratio: early diastolic mitral inflow velocity to early mitral annular velocity ratio; CSA: cardiomyocyte cross-sectional area. Data are expressed as the mean ± SEM. Bold numbers represent the significant effects that were considered. *IxR: when interactions are observed, same superscript letters represent differences (p<0.05) in a row (a = IR0≠SR0; b = IR002≠SR002; c = IR02≠SR02; A = IR0≠IR002; B = IR002≠IR02; C = IR0≠IR02). Sample size: SR0 = 10; SR002 = 10; SR02 = 10; IR0 = 10; IR002 = 8; and IR02 = 9.
(PDF)

S2 Table Energy metabolism and oxidative stress enzymes in Sham and infarcted rats with and without rosemary supplementation. I: infarction; S: Sham; R: Rosemary; R0: no supplementation; R002: 0.02% of rosemary supplementation; R02: 0.2% of rosemary supplementation; LDH activity: lactate dehydrogenase activity (nmol/mg protein); PIDH activity: pyruvate dehydrogenase activity; CS activity: citrate synthase activity; OHADH activity: 3-hydroxyacyl coenzyme-A dehydrogenase activity; ATP sinthase activity; Complex I activity; Complex II activity; LH: lipid hydroperoxide concentration; SOD activity: superoxide dismutase activity; GSH-Px activity: glutathione peroxidase activity. Data are expressed as the mean ± SEM. Bold numbers represent the significant effects that were considered. *IxR: when interactions are observed, same superscript letters represent differences (p<0.05) in a row (a = SR0≠IR0; b = SR002≠IR002; c = IR02≠SR02; A = IR0≠IR002; B = IR002≠IR02; C = IR0≠IR02). Sample size: SR0 = 8; SR002 = 8; SR02 = 8; IR0 = 10; IR002 = 7; and IR02 = 8.
(PDF)

S3 Table Nrf2, HO-1 and PGC1-a expression in Sham and infarcted rats with and without rosemary supplementation by Western blot. I: infarction; S: Sham; R: Rosemary; R0: no supplementation; R002: 0.02% of rosemary supplementation; R02: 0.2% of rosemary supplementation; Nrf-2: nuclear erithroid factor 2; HO-1: heme-oxygenase-1; PGC1α: peroxisome proliferator-activated receptor-α coactivator. Data are expressed as the mean ± SEM. Bold numbers represent the significant effects that were considered. Sample size: SR0 = 10; SR002 = 10; SR02 = 10; IR0 = 10; IR002 = 8; and IR02 = 9.
(PDF)

S4 Table TIMP-1, MMP-2 activity, cytokines and apoptosis markers in Sham and infarcted rats with and without rosemary supplementation. I: infarction; S: Sham; R: Rosemary; R0: no supplementation; R002: 0.02% of rosemary supplementation; R02: 0.2% of rosemary supplementation; PFK activity: Phosphofructokinase activity; TIMP-1: Metaloprotease inhibitor-1; MMP-2: metaloprotease-2; IL-10: interleukin-10; ICAM-1: intercellular adhesion mollecule-1; TNF-α: tumor necrosis factor-α; INF-γ: interferon-γ. Data are expressed as mean ± SEM. Bold numbers represents significant effects considered. Sample size: SR0 = 10; SR002 = 10; SR02 = 10; IR0 = 10; IR002 = 8; and IR02 = 9.
(PDF)
