# Supplementary figures and images for: Rosemary supplementation (Rosmarinus oficinallis L.) attenuates cardiac remodeling after myocardial infarction in rats
Source: PLoS One. 2017 May 11;12(5):e0177521. doi: 10.1371/journal.pone.0177521 (PMC5426768; doi:10.1371/journal.pone.0177521)

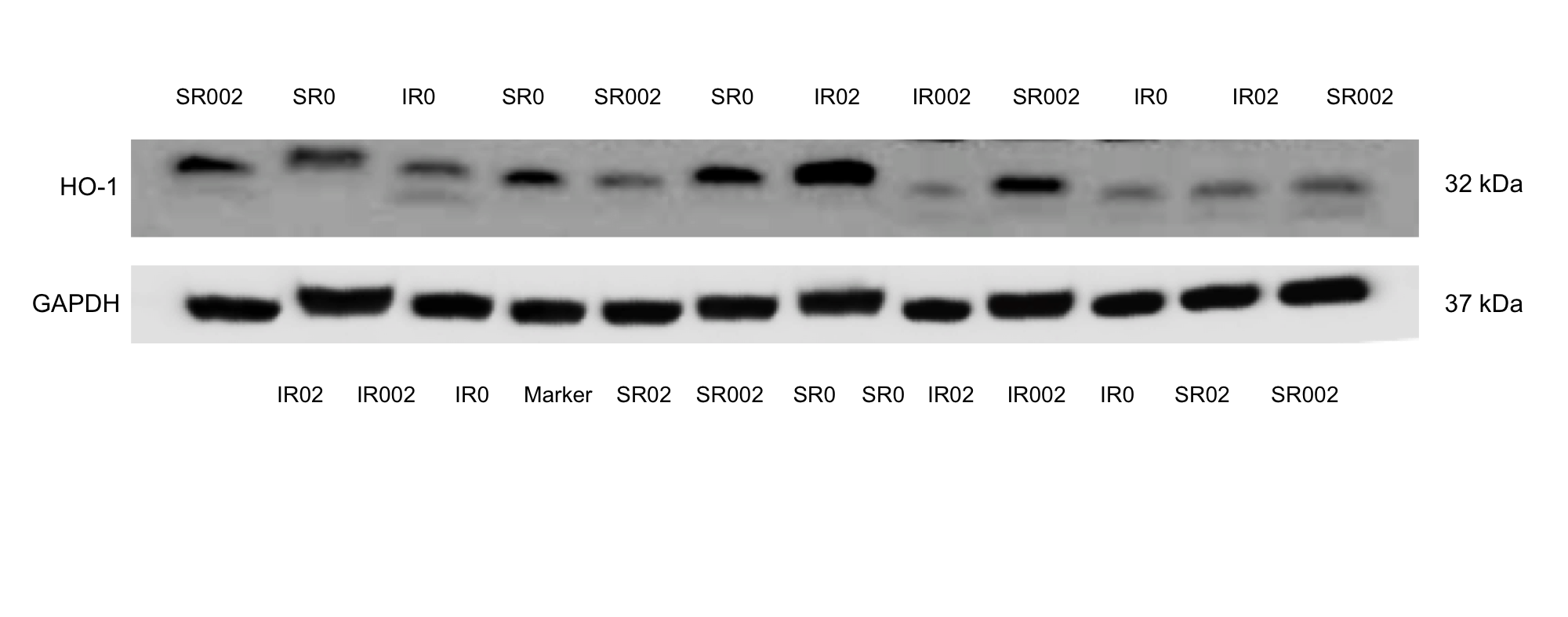

Supplement: S1 Fig — I: infarction; S: Sham; R: Rosemary; R0: no supplementation; R002: 0.02% of rosemary supplementation; R02: 0.2% of rosemary supplementation. Sample size: SR0 = 10; SR002 = 10; SR02 = 10; IR0 = 10; IR002 = 8; and IR02 = 9. (TIF) [file pone.0177521.s001.tif]

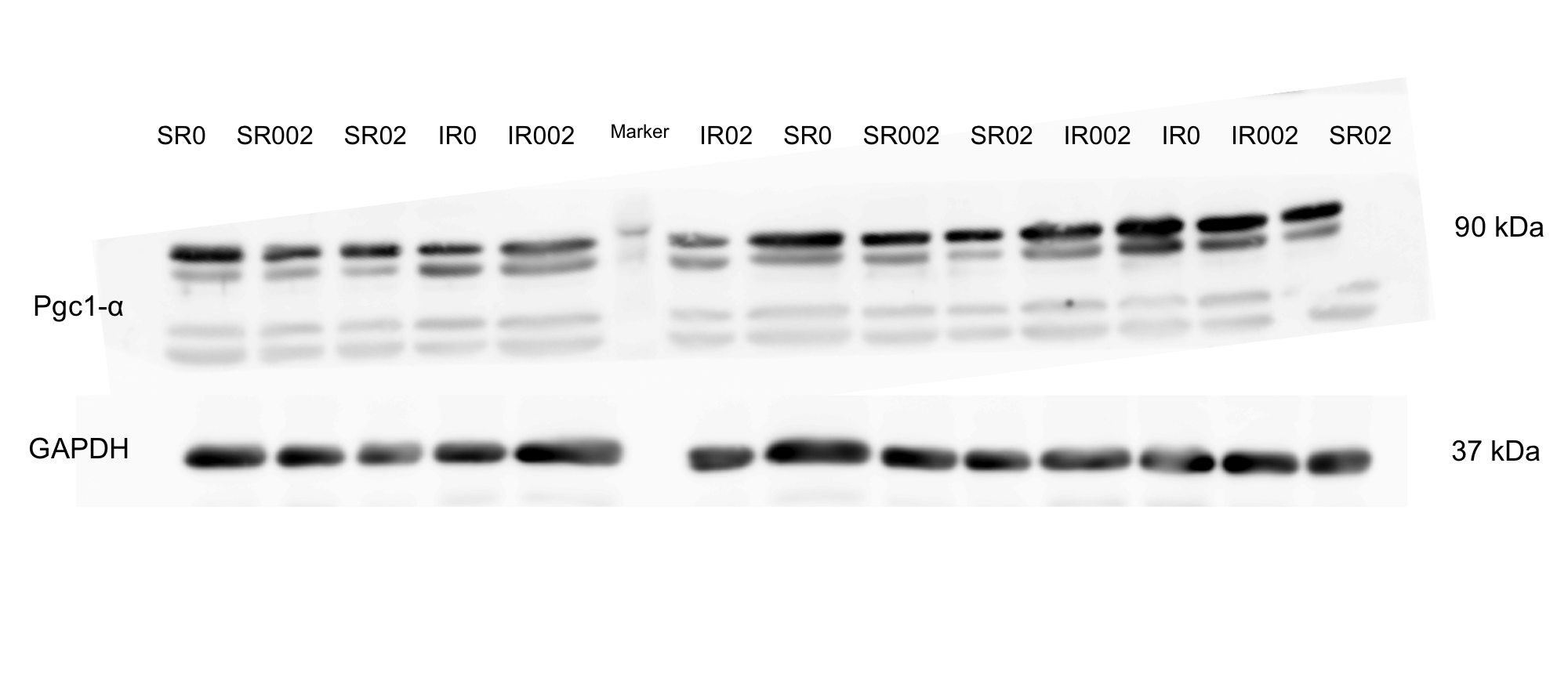

Supplement: S2 Fig — I: infarction; S: Sham; R: Rosemary; R0: no supplementation; R002: 0.02% of rosemary supplementation; R02: 0.2% of rosemary supplementation. Sample size: SR0 = 10; SR002 = 10; SR02 = 10; IR0 = 10; IR002 = 8; and IR02 = 9. (TIF) [file pone.0177521.s002.tif]

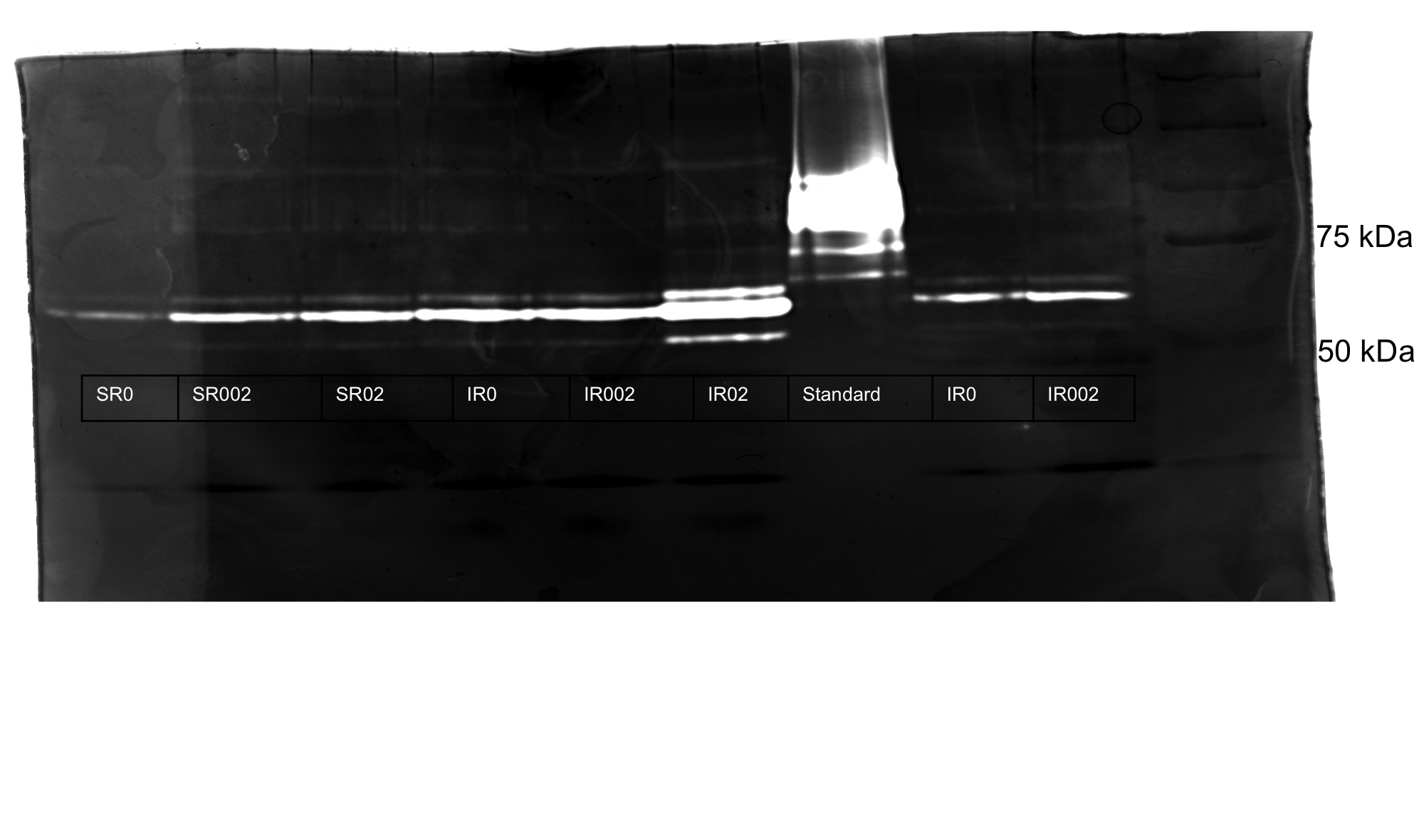

Supplement: S3 Fig — I: infarction; S: Sham; R: Rosemary; R0: no supplementation; R002: 0.02% of rosemary supplementation; R02: 0.2% of rosemary supplementation. Sample size: SR0 = 10; SR002 = 10; SR02 = 10; IR0 = 5; IR002 = 5; and IR02 = 4. (TIF) [file pone.0177521.s003.tif]

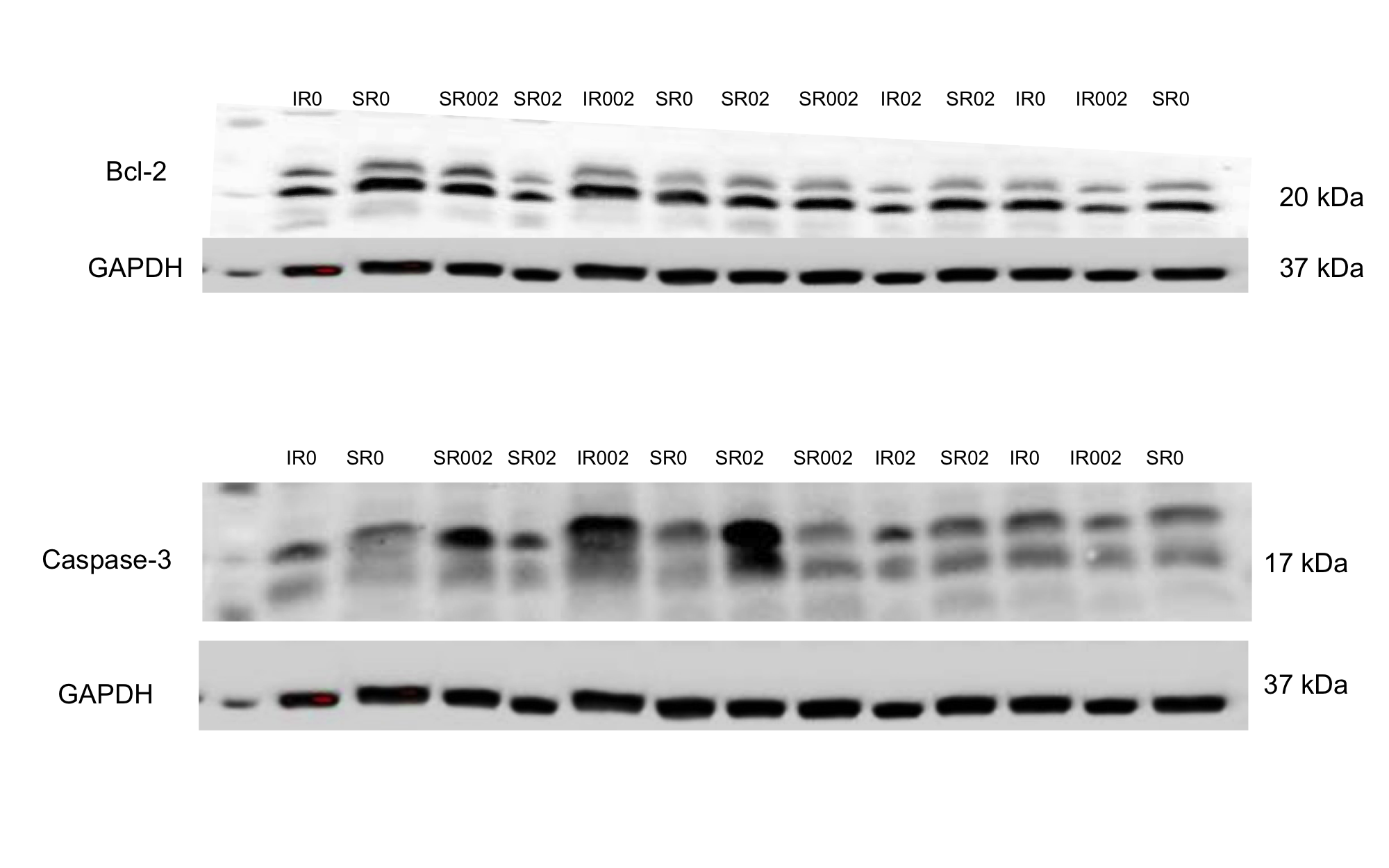

Supplement: S4 Fig — I: infarction; S: Sham; R: Rosemary; R0: no supplementation; R002: 0.02% of rosemary supplementation; R02: 0.2% of rosemary supplementation. Sample size: SR0 = 10; SR002 = 10; SR02 = 10; IR0 = 10; IR002 = 8; and IR02 = 9. (TIF) [file pone.0177521.s004.tif]
