# Supplementary material for: Rosemary supplementation (Rosmarinus oficinallis L.) attenuates cardiac remodeling after myocardial infarction in rats
Source: PLoS One. 2017 May 11;12(5):e0177521. doi: 10.1371/journal.pone.0177521 (PMC5426768; doi:10.1371/journal.pone.0177521)
Supplement: S1 Table — I: infarction; S: Sham; R: Rosemary; R0: no supplementation; R002: 0.02% of rosemary supplementation; R02: 0.2% of rosemary supplementation; BW: body weight; LVDD/BW: left ventricular diastolic diameter indexed for body weight; AO: aortic diameter; LA: left atrial diameter; LA/BW: left atrial diameter indexed for body weight; LA/AO: left atrial diameter indexed for aortic diameter; E wave: peak velocity of early ventricular filling; FAC: fractional area change; EDT: E wave deceleration time; E/E’ ratio: early diastolic mitral inflow velocity to early mitral annular velocity ratio; CSA: cardiomyocyte cross-sectional area. Data are expressed as the mean ± SEM. Bold numbers represent the significant effects that were considered. *IxR: when interactions are observed, same superscript letters represent differences (p<0.05) in a row (a = IR0≠SR0; b = IR002≠SR002; c = IR02≠SR02; A = IR0≠IR002; B = IR002≠IR02; C = IR0≠IR02). Sample size: SR0 = 10; SR002 = 10; SR02 = 10; IR0 = 10; IR002 = 8; and IR02 = 9. (PDF) [file pone.0177521.s005.pdf]

	SHAM groups			Myocardial infarction groups			p values		
	SR0	SR002	SR02	IR0	IR002	IR02	p (I)	p (R)	p (IxR)
**Food intake (g)**	25.8±0.2	24.6±0.2	25.7±0.2	25.4±0.2	25.0±0.2	25.5±0.2	0.187	0.101	0.143
**Infarction size (%)**	-	-	-	41.9±4.5	40.1±4.2	43.6±4,7	-	0.244	-
**Weight gain (g)**	143±8.2	157±8.0	148±8.6	152±8.8	156±8.8	159±10	0.385	0.552	0.773
**LVDD/BW (mm/kg)**	17.9±0.41	17.1±0.39	17.9±0.29	24.6±0.74	24.2±0.68	24.1±0.93	**<0.001**	0.504	0.582
**LA/BW (mm/kg)**	12.2±0.3[a]	12.2±0.5[b]	12.4±0.4[c]	17.3±0.6[a,A]	15.5±1.0[b,A,B]	19.4±0.9[c,B]	<0.001	0.030	**0.001***
**LA/AO**	1.32±0.02[a]	1.41±0.04[b]	1.38±0.03[c]	2.06±0.09[a,A]	1.93±0.14[b.A.B]	2.25±0.08[c,B]	<0.001	0.079	0.024*
**E wave (cm/s)**	75.7±1.5	77.2±2.1	78.9±1.8	98.4±5.5	87.5±8.5	92.7±9.4	**0.014**	0.482	0.246
**Diastolic área (mm^2)**	44.4±1.6	44.5±1.8	43.3±1.6	92.2±3.9	91.7±6.9	85.2±4.5	**<0.001**	0.359	0.557
**FAC (%)**	73.3±0.9	74.3±1.2	75.6±1.0	27.0±2.3	28.2±1.5	27.2±3.5	**<0.001**	0.697	0.757
**Ejection fraction**	0.91±0.01	0.92±0.01	0.93±0.01	0.47±0.02	0.49±0.02	0.46±0.02	**<0.001**	0.571	0.612
**EDT (ms)**	41.7±1.2[a]	44.8±1.9	45.3±1.4[c]	36.1±2.5[a,A]	46.4±2.7[A,B]	31.5±2.0[c,A,B]	0.001	0.002	**0.004***
**E/E' ratio**	19.1±0.6	19.6±0.9	18.8±0.6	24.4±1.7	25.6±2.8	23.6±2.6	**<0.001**	0.898	0.523
**CSA (μm^2)**	176±10[a]	155±11[b]	200±11[c]	285±11[a,A]	232±13[b,A]	265±12[c]	<0.001	<0.001	**<0.001***
**% collagen**	3.97±0.4[a]	3.87±0.8	3.71±0.3	8.73±1.7[a]	5.11±0.4	5.97±0.3	0.046	0.553	**0.035**
