# Supplementary material for: Rosemary supplementation (Rosmarinus oficinallis L.) attenuates cardiac remodeling after myocardial infarction in rats
Source: PLoS One. 2017 May 11;12(5):e0177521. doi: 10.1371/journal.pone.0177521 (PMC5426768; doi:10.1371/journal.pone.0177521)
Supplement: S2 Table — I: infarction; S: Sham; R: Rosemary; R0: no supplementation; R002: 0.02% of rosemary supplementation; R02: 0.2% of rosemary supplementation; LDH activity: lactate dehydrogenase activity (nmol/mg protein); PIDH activity: pyruvate dehydrogenase activity; CS activity: citrate synthase activity; OHADH activity: 3-hydroxyacyl coenzyme-A dehydrogenase activity; ATP sinthase activity; Complex I activity; Complex II activity; LH: lipid hydroperoxide concentration; SOD activity: superoxide dismutase activity; GSH-Px activity: glutathione peroxidase activity. Data are expressed as the mean ± SEM. Bold numbers represent the significant effects that were considered. *IxR: when interactions are observed, same superscript letters represent differences (p<0.05) in a row (a = SR0≠IR0; b = SR002≠IR002; c = IR02≠SR02; A = IR0≠IR002; B = IR002≠IR02; C = IR0≠IR02). Sample size: SR0 = 8; SR002 = 8; SR02 = 8; IR0 = 10; IR002 = 7; and IR02 = 8. (PDF) [file pone.0177521.s006.pdf]

	**SHAM groups**			**Myocardial infarction groups**			**p values**		
	SR0	SR002	SR02	IR0	IR002	IR02	p (I)	p (R)	p (IxR)
**LDH activity (nmol/mg protein)**	162±8.0^{a}	179±11.8^{b}	165±8.0	192±11.8^{a,A,C}	147±9.6^{c,A}	151±8.0^{C}	0.525	0.063	**0.014***
**PIDH activity (nmol/mg tissue)**	323±12.9	349±17.2	342±8.50	275±14.3	327±14.9	319±4.88	**0.041**	**0.062**	0.489
**CS activity (nmol/mg tissue)**	26.1±1.5^{a}	26.2±1.7^{b}	26.6±2.3	18.3±1.2^{a,A,C}	34.8±3.5^{b,A}	31.2±1.2^{C}	0.152	0.011	**<0.001***
**OHADH activity (nmol/mg protein)**	22.9±2.5	18.5±2.7^{b}	16.4±1.7^{c}	32.2±2.5^{A,C}	58.6±6.5^{b,A}	59.8±7.4^{c,C}	<0.001	0.071	**<0.001***
**ATP sinthase activity (nmol/mg tissue)**	24.7±1.9	27.8±2.5	34.6±3.7	26.7±2.1	36.3±3.2	38.5±2.8	**0.039**	**0.008**	0.569
**Complex I activity (nmol/mg tissue)**	3.8±0.2^{a}	3.5±0.4^{b}	3.8±0.3	2.8±0.3^{a,A,C}	4.6±0.5^{b,A}	4.4±0.2^{C}	0.389	0.03	**0.004***
**Complex II activity**	6.0±0.4	6.2±0.4	5.6±0.5^{c}	5.4±0.2^{C}	5.5±0.5^{B}	8.3±0.5 ^{c,B,C}	0.192	0.009	**<0,001***

**(nmol/mg tissue)**									
**LH (nmol/mg tissue)**	202±10.2^{a}	233 ±9.8^{b}	230±10.3	331±12.7^{a,C}	319±9.6^{b,A,B,C}	235±9.6^{B,C}	<0.001	<0.001	**<0.001***
**Catalase activity (nmol/mg tissue)**	92.4±7.0	79.2±12.7	92.1±5.6	67.3±4.3	87.9±5.8	77.5±4.3	0.091	0.766	0.072
**SOD activity (nmol/mg protein)**	7.1±0.8^{a}	8.5±0.7	8.5±0.9	13±0.6^{a,A,C}	8.9±0.3^{A}	8.7±0.2^{C}	<0.001	0.274	**<0.001***
**GSH-Px activity (nmol/mg tissue)**	34±1.3^{a}	29±1.4	29±3.0	19±0.6^{a}	24±3.2	28±3.1	<0.001	0.712	**0.031***
