# Supplementary material for: Rosemary supplementation (Rosmarinus oficinallis L.) attenuates cardiac remodeling after myocardial infarction in rats
Source: PLoS One. 2017 May 11;12(5):e0177521. doi: 10.1371/journal.pone.0177521 (PMC5426768; doi:10.1371/journal.pone.0177521)
Supplement: S3 Table — I: infarction; S: Sham; R: Rosemary; R0: no supplementation; R002: 0.02% of rosemary supplementation; R02: 0.2% of rosemary supplementation; Nrf-2: nuclear erithroid factor 2; HO-1: heme-oxygenase-1; PGC1α: peroxisome proliferator-activated receptor-α coactivator. Data are expressed as the mean ± SEM. Bold numbers represent the significant effects that were considered. Sample size: SR0 = 10; SR002 = 10; SR02 = 10; IR0 = 10; IR002 = 8; and IR02 = 9. (PDF) [file pone.0177521.s007.pdf]

	**SHAM groups**			**Myocardial infarction groups**			**p values**		
	SR0	SR002	SR02	IR0	IR002	IR02	p (I)	p (R)	p (IxR)
**Nrf-2 expression (arbitrary unit)**	1.61±0.1	1.23±0.2	1.01±0.4	0.82±0.8	1.09±0.6	0.66±0.5	**0.050**	0.336	0.448
**HO-1 expression (arbitrary unit)**	0.71±0.2	0.55±0.2	0.52±0.2	0.43±0.2	0.47±0.2	0.36±0.2	0.254	0.742	0.853
**PGC1α expression (arbitrary unit)**	0.55±0.7	0.63±0.7	0.78±0.5	1.17±0.7	0.80±0.4	0.84±0.7	0.208	0.868	0.543
