# Supplementary material for: Rosemary supplementation (Rosmarinus oficinallis L.) attenuates cardiac remodeling after myocardial infarction in rats
Source: PLoS One. 2017 May 11;12(5):e0177521. doi: 10.1371/journal.pone.0177521 (PMC5426768; doi:10.1371/journal.pone.0177521)
Supplement: S4 Table — I: infarction; S: Sham; R: Rosemary; R0: no supplementation; R002: 0.02% of rosemary supplementation; R02: 0.2% of rosemary supplementation; PFK activity: Phosphofructokinase activity; TIMP-1: Metaloprotease inhibitor-1; MMP-2: metaloprotease-2; IL-10: interleukin-10; ICAM-1: intercellular adhesion mollecule-1; TNF-α: tumor necrosis factor-α; INF-γ: interferon-γ. Data are expressed as mean ± SEM. Bold numbers represents significant effects considered. Sample size: SR0 = 10; SR002 = 10; SR02 = 10; IR0 = 10; IR002 = 8; and IR02 = 9. (PDF) [file pone.0177521.s008.pdf]

	SHAM Groups			Myocardial infarction groups			p values		
	SR0	SR002	SR02	IR0	IR002	IR02	p (I)	p (R)	p (IxR)
**PFK activity (nmol/mg tissue)**	117±7.8	121±7.5	115±9.3	129±10.9	116±6.8	108±4.2	0.988	0.677	0.729
**TIMP-1 (pg/mg protein)**	84.2±10.6	98.2±24.7	127±31.2	113±41.5	70.3±16.0	112±3.5	0.259	0.78	0.549
**Active MMP-2 (arbitrary unit)**	13±4.3	7.8±3.1	13.8±3.7	29.1±15.0	9.6±4.6	58.6±42.1	0.181	0.228	0.986
**Intermediate MMP-2 (arbitrary unit)**	169±23	195±12	207±8	218±11	214±19	225±8	**0.032**	0.360	0.504
**Total MMP-2 (arbitrary unit)**	243±52	242±19	269±15	333±45	274±37	402±96	**0.033**	0.266	0.572
**Active/Total MMP-2**	0.05±0.001	0.03±0.01	0.05±0.01	0.07±0.02	0.03±0.01	0.10±0.06	0.346	0.232	0.940
**IL-10 (pg/mg protein)**	46.9±4.2	45.5±5.9	63.9±10.5	32.6±5.8	34.2±7.9	23.9±2.5	**0.002**	0.826	0.149
**ICAM-1 (pg/mg protein)**	95.7±10.2	95.1±7.8	115±15.6	92.1±1.4	99.3±22.6	74.7±11.7	0.224	0.969	0.214
**TNF-α (pg/mg protein)**	41.8±4.2	41.2±5.6	58.3±10.5	26.8±3.1	27.9±5.8	18.4±3.5	**<0.001**	0.823	0.146

**INF-γ (pg/mg protein)**	28.9±2.6	27.6±3.4	36.3±5.9	19.8±3.0	20.9±4.1	15.5±1.8	**0.002**	0.913	0.246
**Caspase-3 (arbitrary unit)**	0.47±0.71	0.55±0.35	0.52±0.12	0.71±0.33	0.47±0.12	0.52±0.22	0.254	0.742	0.853
**Bcl-2 (arbitrary unit)**	1.30±0.47	0.94±0.38	0.80±0.24	0.65±0.31	0.91±0.45	0.41±0.22	**0.046**	0.216	0.416
